# Concurrent validity of the Swedish version of the life-space assessment questionnaire

**DOI:** 10.1186/s12877-016-0357-4

**Published:** 2016-11-08

**Authors:** Sofi Fristedt, Ann-Sofi Kammerlind, Marie Bravell Ernsth, Eleonor I. Fransson

**Affiliations:** 1 Jönköping University, School of Health and Welfare, Box 1026, SE-551 11 Jönköping, Sweden; 2 Futurum – the Academy for Healthcare, Region Jönköping County, SE-551 85 Jönköping, Sweden; 3 Department of Medical and Health Sciences, Linköping University, SE-581 83 Linköping, Sweden

**Keywords:** Activities, Mobility, Older persons, Validity

## Abstract

**Background:**

The Life-Space Assessment (LSA), developed in the USA, is an instrument focusing on mobility with respect to reaching different areas defined as life-spaces, extending from the room where the person sleeps to mobility outside one’s hometown. A newly translated Swedish version of the LSA (LSA-S) has been tested for test-retest reliability, but the validity remains to be tested. The purpose of the present study was to examine the concurrent validity of the LSA-S, by comparing and correlating the LSA scores to other measures of mobility.

**Method:**

The LSA was included in a population-based study of health, functioning and mobility among older persons in Sweden, and the present analysis comprised 312 community-dwelling participants. To test the concurrent validity, the LSA scores were compared to a number of other mobility-related variables, including the Short Physical Performance Battery (SPPB) as well as “stair climbing”, “transfers”, “transportation”, “food shopping”, “travel for pleasure” and “community activities”. The LSA total mean scores for different levels of the other mobility-related variables, and measures of correlation were calculated.

**Results:**

Higher LSA total mean scores were observed with higher levels of all the other mobility related variables. Most of the correlations between the LSA and the other mobility variables were large (*r* = 0.5–1.0) and significant at the 0.01 level. The LSA total score, as well as independent life-space and assistive life-space correlated with transportation (0.63, 0.66, 0.64) and food shopping (0.55, 0.58, 0.55). Assistive life-space also correlated with SPPB (0.47). With respect to maximal life-space, the correlations with the mobility-related variables were generally lower (below 0.5), probably since this aspect of life-space mobility is highly influenced by social support and is not so dependent on the individual’s own physical function.

**Conclusion:**

LSA was shown to be a valid measure of mobility when using the LSA total, independent LS or assistive LSA.

## Background

In later life, the risk of experiencing limited mobility is increased [1], with limited activity in daily life and restricted participation in social life as a consequence [2, 3]. This risk is evident in Sweden and other Western countries despite general good accessibility in public environments and in housing [4], in cities as well as more rural areas. In order to guide and evaluate mobility-related interventions, it is essential to gain more knowledge about older people’s mobility, and factors related to mobility limitations. For these purposes valid instruments to measure mobility are vital. Most measurements of mobility used for clinical and research purposes focus on performance at a specific point of time or other aspects such as mobility-related tiredness [4]; mobility limited to walking [5]; mobility as an aspect of personal or instrumental Activities of Daily Living (ADL) [6]; or environmental aspects influencing personal mobility [7]. Unlike these instruments, the Life-Space Assessment (LSA), developed in the USA, is an instrument focusing on mobility with respect to reaching different areas defined as life-spaces extending from the room where the person sleeps, to mobility outside one’s hometown [8, 9]. In addition, this instrument goes beyond measuring the mobility a person is capable of a certain day by considering attained life-spaces during the previous month, The LSA also takes into consideration the frequency and independence of a person’s mobility [10, 11]. The LSA has been used to measure mobility in different populations, for example community-dwelling older adults [12], people with eye diseases [13], wheelchair users [1], power mobility device users [14], older people after hospitalization [15], and palliative care patients [16]. The questionnaire has previously been translated, e.g. into French-Canadian [14], Finnish [17, 18], Japanese [19], Spanish [20, 21], and Portuguese versions [20].

Versions translated to native languages are needed to ensure standardised measurements across settings. These versions need to be tested to determine the reliability and validity of the measurements. A newly translated Swedish version of The LSA has been tested for test-retest reliability, with the ratings consistent between the two test occasions for all four LSA scoring methods (total LSA, independent, assistive and maximal life-space scores) [22].

The validity of the original version [23] as well as the Portuguese and Spanish versions [20] has been demonstrated, but the validity of the Swedish version of the LSA is yet to be tested. The purpose of the present study was to examine the concurrent validity of the Swedish version of LSA (LSA-S), by comparing LSA-scores to other measures of mobility.

## Method

### Participants and data collection

The LSA was included in a population-based study of health, functioning and mobility among older persons in Sweden. The study population was randomly selected from a population register. Men and women, 75, 80, 85 or 90 years-old, living in Jönköping County Council, Sweden, in 2009 and 2010, were eligible. Similar to the rest of Sweden (and other Western countries), Jönköping County is considered as accessible in terms of public environments and housing. Persons for whom we had information from relatives or staff that they suffered from dementia, were excluded from the study. In total, 327 persons voluntarily chose to participate and gave their informed written consent. The study was approved by the regional ethical committee in Linköping, Sweden (#225-08). Data collection, including interviews and tests of physical performance, was performed by trained nurses during visits to participants’ homes. During the interview, the participants responded to the LSA and a large set of other questions. Physical performance was assessed by the Short Physical Performance Battery (SPPB) [24]. The present study excluded subjects living in nursing homes (*n* = 10), and also those who had not been assessed by the LSA *(n* = 5*)* yielding an analytical sample of 312 participants.

### Instruments and variables

The LSA includes six levels of life-space, ranging from the person’s bedroom (Life-space 0) to places beyond the person’s hometown (Life-space 5) [10]. For each of these six levels, the person is asked how often they have been to that specific life-space area during the last 4 weeks, and whether they did so independently or needed assistance from another person or equipment. A total LSA score is obtained by multiplying the life-space level reached (1–5) by the value for the frequency of transportation (1–4) for each life-space level, as well as by value for independence (2, 1.5, or 1) and then summarizing the scores of the five levels. The total score can range from 0 (totally confined to bed) to 120 (independent, with daily out-of-town mobility). Moreover, three additional measures of life-space levels can be calculated. The *independent life-space* level indicates the highest level obtained without any assistance. The *assistive life-space* level defines the highest level reached with help from equipment but not another person. Finally, the *maximal life-space* level indicates the greatest distance travelled irrespective of assistance from equipment and/or another person. These three measures can range from 0 (not leaving the bedroom) to 5 (visiting places outside one’s own town).

To test the concurrent validity of The LSA, seven variables involving mobility or activities dependent on mobility were chosen, namely;

- SPPB, utilised to assess older persons physical performance [24], was used mainly as a “total score” summarizing the three different subtest scores, i.e., “total balance” including a hierarchical test of standing balance, “gait speed” during a 3-m walk and “chair stand” including five repetitive chair stands, ranging from 1 (poor ability) to 12 (good ability).
- Stair climbing, corresponding to the question in SF-12 [27]: Are you limited in your ability to climb several flights of stairs (1 = yes, very limited/2 = yes, somewhat limited/3 = no, not at all limited)?
- Transfers: Are you able to transfer yourself independently out of bed or between two chairs (1 = not able/2 = large problem/3 = some problem/4 = no problem)?
- Transportation: Are you able to transport yourself independently (1 = not able/2 = large problem/3 = some problem/4 = no problem)?
- Food shopping: Are you able to shop for food independently (1 = not able/2 = large problem/3 = some problem/4 = no problem)?
- Travel for pleasure: Do you travel for pleasure (as a leisure activity) (1 = never/2 = less than monthly/3 = monthly/4 = weekly/5 = daily)?
- Community activities: Do you take part in community activities (i.e., meetings, courses, political activities, non-profit work, cultural activities, spectator sport, choir, religious meetings)? An index was created by counting the actual number, within the eight suggested community activities that the participants took part in at least once a month.

### Analysis

Descriptive statistics were used as appropriate, i.e., mean and standard deviations (SD) for normally distributed data (i.e. LSA total score), while median and inter-quartile range (Q1-Q3) were used in all other cases. To analyse concurrent validity, The LSA total mean scores for different levels of the other mobility-related variables were calculated. Moreover, correlations between The LSA total, independent, assistive and maximal life-space, as well as the correlation between these four LSA measures and the other mobility-related variables were calculated using Spearman correlations, since most variables were measured by ordinal scales. Using the guidelines from Cohen [26] a correlation coefficient of 0.50–1.0 was considered large, 0.30–0.49 medium and 0.10–0.29 small.

## Results

In Table 1, the characteristics of the sample as regards background information, life-space mobility and other mobility-related variables are described. Participants were on average 80 years old. Slightly more than half of the participants were married, and the majority were living in a town. The majority did not use any assistive devices for mobility. The participants had an average of 65 (min 8–max 120) on the LSA total score (0–120).

**Table 1:** Characteristics of the study sample (*n* = 312)

Variable	
Sex, *n* (%)	
Males	147 (47)
Females	165 (53)
Age in years, mean (SD)	80 (5)
Marital status, *n* (%)	
Married	164 (53)
Widows/widowers	119 (38)
Never married	13 (4)
Divorced	16 (5)
Type of housing, *n* (%)	
Own house	156 (50)
Apartment	156 (50)
Type of living area, *n* (%)	
Living in town >5000 inhabitants	206 (66)
Living in village 200–5000 inhabitants	68 (22)
Living in small village <200 inhabitants	13 (4)
Living in the countryside, not in village	25 (8)
Use of assistive devices, *n* (%)	
Yes	103 (33)
No	209 (66)
LSA total score (0–120), mean (SD)	65 (23)
Independent life-space (0–5), median (Q1-Q3)	4 (2–5)
Assistive life-space (0–5), median (Q1-Q3)	5 (4–5)
Maximal life-space (0–5), median (Q1-Q3)	5 (5–5)
SPPB total score (0–12), median (Q1-Q3)	10 (7–11)
total balance (0–4), median (Q1-Q3)	4 (3–4)
gait speed (0–4), median (Q1-Q3)	3 (2–4)
chair stand (0–4), median (Q1-Q3)	3 (1–4)
Stair climbing (1–3), median (Q1-Q3)	2 (2–3)
Transfers^a^ (1–4), median (Q1-Q3)	4 (4–4)
Transportation (1–4), median (Q1-Q3)	4 (3–4)
Food shopping (1–4), median (Q1-Q3)	4 (4–4)
Travel for pleasure (1–5), median (Q1-Q3)	2 (1–2)
Community activities (0–8), median (Q1-Q3)	1 (0–2)

^a^transfer independently out of bed or between two chairs

In Table 2, correlations between the four LSA measures are shown. All correlations were significant on the 0.01 level. The strongest correlations were observed between the LSA total score and independent as well as assistive life-space scores, with somewhat lower correlations observed for maximal life-space in relation to the other LSA measures.

**Table 2:** Correlations^a^ between the LSA total score, independence, assistance and maximal life-space

	LSA total	Independent life-space	Assistive life-space	Maximal life-space
LSA total (0–120)	1.00			
Independent life-space (0–5)	0.84^b^	1.00		
Assistive life-space (0–5)	0.79^b^	0.78^b^	1.00	
Maximal life-space (0–5)	0.50^b^	0.45^b^	0.58^b^	1.00

^a^Spearman ^b^significant on 0.01 level

**Fig. 1:**
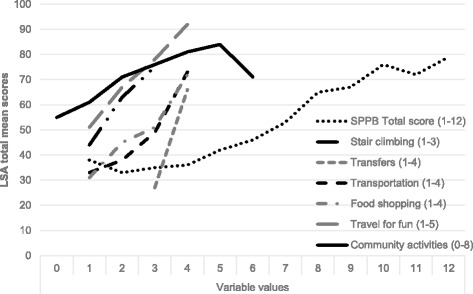
The LSA total mean scores in relation to other mobility-related variables

The LSA total mean scores by levels of other mobility-related variables are displayed in Fig. 1. Similar patterns were observed in all variables, i.e., higher LSA total mean scores were observed with higher levels of the other mobility-related variables. It is worth mentioning that no participants in this sample were unable (1) to independently “transfer” out of bed or between two chairs, or (2) reported great difficulty in doing so. Out of eight suggested community activities, the maximum number that any participant took part in at least once a month were six activities. Moreover, only two persons participated in six “community activities” explaining the drop in that line.

Finally, Table 3 shows correlations between all four LSA measures and the mobility-related variables. Overall, correlations between the LSA and the other mobility-related variables were large, especially for the LSA total and independent and assistive life-space. With respect to maximal life-space, the correlations with other variables were generally lower. The correlations between the SPPB sub-scores, i.e., total balance, gait speed and chair stand, and the LSA measures showed the same pattern as for the SPPB total score (data not shown).

**Table 3:** Correlations^a^ between the LSA and mobility-related variables

	LSA total	Independent life-space	Assistive life-space	Maximal life-space
SPBB total score (0–12)	0.57^b^	0.63^b^	0.47^b^	0.20^b^
Stair climbing (1–3)	0.45^b^	0.42^b^	0.28^b^	0.10
Transfers (1–4)	0.28^b^	0.30^b^	0.28^b^	0.04
Transportation (1–4)	0.63^b^	0.66^b^	0.64^b^	0.26^b^
Food shopping (1–4)	0.55^b^	0.58^b^	0.55^b^	0.29^b^
Travel for pleasure (1–5)	0.42^b^	0.44^b^	0.41^b^	0.20^b^
Community activities (0–8)	0.38^b^	0.35^b^	0.35^b^	0.17^b^

^a^Spearman, ^b^significant on 0.01 level

## Discussion

In the present study the concurrent validity of the Swedish version of the LSA (LSA-S) was examined by comparing the LSA-scores to other measures of mobility. Most correlations between the different LSA measures and the other mobility-related variables were large (above 0.50). Thus, in terms of concurrent validity, thus LSA was shown to be a valid measure of mobility in a Swedish setting among community-dwelling older persons.

Measures reflecting aspects of mobility that presumably could be related to the LSA were chosen. In fact, as far as we know, there is no golden standard described in the literature that could be used to assess concurrent validity related to assessment of life-space mobility. This could be considered a limitation in the present study. However, previous studies on the validity of the LSA have, similarly to our study, used measures of physical performance, including SPPB [20], and ADL [20], or have focused on associations between physical activity and life-space mobility [18]. In addition, our study also considered participation in community activities since that may reflect life-space mobility. Similar to our study, these previous studies found evidence for validity of the LSA by observing significant correlations between the LSA and other mobility-related variables. In fact, most correlations between the different the LSA measures and the other mobility-related variables were also large (above 0.50) in previous studies [20]. Moreover, similar levels of positive correlations were found between physical activity and life-space mobility in a recent study [18].

As previously shown [10], correlations between the different LSA scores were large overall with respect to LSA total, independent and assistive life-space (0.78–0.84), but lower between maximal life-space and the other three LSA scores (0.45–0.58). This may be due to the ceiling effect of maximal life-space found in this study, with most of the study participants reaching the highest score possible. Maximal life-space includes compensation by both persons and equipment, whereas the other LSA scores only consider the persons’ own ability, or in the case of assistive life-space, with the assistance of equipment but without help of another person. With the help of both another person and equipment the person’s own ability is highly compensated, potentially creating this ceiling effect of the maximal life-space score. Thus maximal life-space includes somewhat different aspects of mobility, for example social support [10], than LSA total and independent as well as assistive life-space, which may explain the low correlations. Low correlations were also found between maximal life-space and the other mobility-related variables. In addition to the narrow range of values on the maximal life-space in this study sample, these low correlations may also be due to the fact that several of the measures (unlike maximal life-space), focused on independent performance. Overall, independent life-space probably best reflects the chosen mobility-related variables compared to the other LSA scores. Not surprisingly, this was especially true for “SPPB total score”, “transfers” and “transportation”. In fact, the two last (“transfers” and “transportation”), were based on independent performance.

The mobility-related variables that were analysed in relation to LSA in this study included different aspects of mobility, ranging from more functional aspects, as in SPPB, through more activity-related issues, as in “stair climbing” and the other ADL activities, to also include aspects of participation as in the “community activities” variable. If the environment is supportive, as in Swedish and other Western context, and other alternatives are available, stair climbing is not a prerequisite for community mobility, and this may offer one explanation for the lower correlations with the LSA for that variable. Generally low correlations were found related to “transfers”, potentially due to the fact that values were observed in only the two highest categories of this variable in our sample. It is worth mentioning that low values for this variable may indicate substantial mobility problems, but such problems were not evident in this sample of community-dwelling older adults. Generally, SPPB and “transportation” achieved the highest correlations with the LSA scores. Both SPPB and “transportation” are likely to reflect functional and activity-related factors that support or indicate life-space mobility, while some of the other variables (“travel for fun” and “community activities”) not only indicate life-space mobility but also elements of participation such as motivation, engagement and opportunities.

Comparing the LSA total scores with other mobility-related variables indicated consistency between measures, i.e., higher means of LSA total score also meant an increase in ability as measured by other mobility-related variables. Similar to the previous study on the LSA test-retest reliability [22] we recommend using the total score since that seems to give the most complete picture of mobility compared to the three separate life-space levels.

Finally, it may be considered a limitation that not all variables, i.e., “transfers”, “transportation” “food shopping”, “travel for pleasure” and “community activities”, were part of a standardised test. However, these variables have been used in similar surveys by the research group before, and found to work well in similar contexts. “Travel for pleasure” and “community activities” were based on self-reported, time-based frequency of participation (daily to never). The items of “transfers”, “transportation” and “food shopping” were self-rated by the participants using the ordinal scale (1 = not able/2 = large problem/3 = some problem/4 = no problem). The participants were not given any further definition of these values, so their rating were subjective. This may be considered a limitation, but could be motivated by the argument that mobility is more than moving from A to B and thus not only affected by objective conditions, but also influenced by previous and current subjective feelings and individual experiences [25]. It should also be noted that the variable of “transportation” includes walking as well as transport mobility including various vehicles.

Another limitation relates to the rather high levels of especially assistive and maximum life-space indicating a ceiling effect of these measures, or similar to many other gerontological studies, that we have identified a rather healthy sample.

## Conclusion

LSA was shown to be a valid measure of mobility when using the LSA total, independent LS or assistive LSA.

## Abbreviations

LSA: Life-space assessment
SPPB: Short physical performance battery
